# Corrigendum to: Impact of reference change value (RCV) based autoverification on turnaround time and physician satisfaction

**DOI:** 10.11613/BM.2017.031203

**Published:** 2017-10-15

**Authors:** Esther Fernández-Grande, Carolina Varela-Rodriguez, Luis Sáenz-Mateos, Amparo Sastre-Gómez, Pilar García-Chico, Teodoro J. Palomino-Muñoz

**Affiliations:** 1 Servicio de Análisis Clínicos, Hospital General Universitario de Ciudad Real, Ciudad Real, Spain.; 2 Servicio de Medicina Preventiva, Hospital General Universitario de Ciudad Real, Ciudad Real, Spain.; 3 Servicio de Análisis Clínicos, Complejo Hospitalario de Navarra, Pamplona, Spain

This is a correction for *Biochemia Medica* 2017;27(2): 342-49. DOI: https://doi.org/10.11613/BM.2017.037.

Since the publication of this article, the authors have noticed that the surname of the second author in the byline was published incorrectly. The correct byline is presented above. The authors apologize for any inconvenience caused to the readers.

